# Ab Initio Chemical Kinetics for Self- and Cross-Reactions of *anti*- and *syn*-CH_3_CHOO Conformers

**DOI:** 10.1021/acs.jpca.6c00127

**Published:** 2026-03-17

**Authors:** Hue-Phuong Trac, Putikam Raghunath, Ming-Chang Lin

**Affiliations:** Department of Applied Chemistry and Center for Emergent Functional Matter Science, 34914 National Yang Ming Chiao Tung University, Hsinchu 300093, Taiwan

## Abstract

The mechanisms for the self- and cross-reactions of *anti*-CH_3_CHOO and *syn*-CH_3_CHOO conformers have been investigated by ab initio quantum-chemical and statistical-theory calculations. The results of the study indicate that at 298 K under 5–Torr He pressure, the self-reaction of *anti*-CH_3_CHOO is the fastest with *k*_aa_ = 4.90 × 10^–10^ cm^3^ molecule^–1^ s^–1^, the *anti-syn* cross-reaction with *k*_as_ = 1.82 × 10^–10^ cm^3^ molecule^–1^ s^–1^, and the self-reaction of *syn*-CH_3_CHOO with *k*_ss_ = 1.28 × 10^–10^ cm^3^ molecule^–1^ s^–1^. The theoretical results, including the deactivation of internally excited dimers formed by initial bimolecular association reactions accounting for more than 50% of the predicted rates, agree with the recent experimental data within reported errors measured at 298 K and 2–10 Torr He pressure.

## Graphical abstract

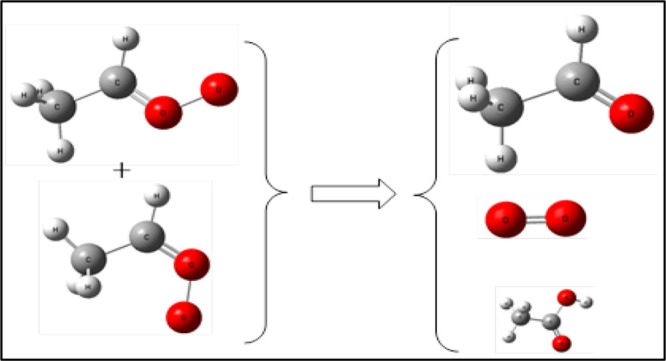

## 1 Introduction

The generation and detection of small CIs (CH_2_OO and CH_3_CHOO), as well as their reaction kinetics with pollutants (NO_*x*_ and SO_2_) have been investigated.^1–5^ Under higher concentration conditions, a CI molecule can undergo a very rapid bimolecular self-reaction, as first demonstrated experimentally and theoretically by Su et al.^4^ for the CH_2_OO case, thanks to its zwitter-ionic character which helps facilitate the head-to-tail bimolecular interaction. As CH_3_CHOO has two structural isomers, *syn*- and *anti*-CH_3_CHOO, one would expect a much more complicated situation, according to the very recent study by Kao et al.^3^ employing a new IR/UV dual-probe multipass absorption system to study the production of *syn*- and *anti*-CH_3_CHOO conformers and measure the kinetics of their self- and cross-reactions.

In the study of Kao et al.,^3^ the branching ratio for the formation of the *syn*- and *anti*-CH_3_CHOO conformers from the CH_3_CHI + O_2_ reaction was reported to be (80 ± 10): (20 ± 10) at 298 K under 5–Torr He pressure. The stereospecific product ratio and/or the absolute rate constants for their production reported by Kao et al.^3^ and others^1,6,7^ near room temperature under similar He pressures, could be quantitatively accounted for by our quantum-statistical theory calculations.^9^ Furthermore, as alluded to above, Kao et al.^3^ also determined the self- and cross-reaction rate constants of the CH_3_CHOO conformers. The rate constants for the *anti*-CH_3_CHOO + *anti*-CH_3_CHOO, *anti*-CH_3_CHOO + *syn-*CH_3_CHOO, and *syn*-CH_3_CHOO + *syn*-CH_3_CHOO reactions at 298 K were reported to be *k*_aa_ = (6 ± 2) × 10^–10^ cm^3^ molecule^–1^ s^–1^, *k*_as_ = (2.1 ± 0.6) × 10^–10^ cm^3^ molecule^–1^ s^–1^, and *k*_ss_ = (1.4 ± 0.3) × 10^–10^ cm^3^ molecule^–1^ s^–1^, respectively.

The near gas-kinetic rate constants for the CH_3_CHOO conformer reactions given above, similar to the CH_2_OO case with the reported rate constant, (4.1 ± 0.8) × 10^–10^ cm^3^ molecule^–1^ s^–1^ at 343 K,^4^ are attributable to the zwitter-ionic nature of the CIs with the >C^+^OO^–^ charge distribution. In the CH_2_OO case, the zwitter-ionic property allows the barrierless association of 2 CH_2_OO molecules to occur with the terminal O atom of one CH_2_OO molecule binding with the C atom of another CH_2_OO molecule, forming a 6-membered-ring (CH_2_OO)_2_ dimer, which rapidly fragments giving 2 CH_2_O + O_2_ (^1^Δ) exothermically.^4^

In the present study, we investigate the mechanisms responsible for the self- and cross-reactions of the CH_3_CHOO conformers with the reported, much different kinetics, *k*_aa_ > *k*_as_ > *k*_*ss*_, by quantum-statistical theory calculations.

## 2 Computational Methods

### 2.1 Ab Initio Calculations

The electronic structures of all the species involved in the self- and cross-reactions of CH_3_CHOO were optimized at the B3LYP/aug-cc-pVTZ level.^8–10^ The vibrational frequencies were also computed at the same level. The final energies were computed at the CCSD­(T)/aug-cc-pVTZ level based on the optimized structures.^11^ All the calculations were carried out using the Gaussian 16 program package.^12^

### 2.2 Rate Constant Predictions

The kinetics of the bimolecular reactions of CH_3_CHOO conformers were studied using the transition state theory (TST)^13^ and the Rice-Ramsperger-Kassel-Marcus (RRKM) theory.^14^ For a simple reaction not involving a long-lived intermediate with a well-defined transition state, TST was employed to predict its rate constant. For the reaction steps with barrierless reaction channels, such as *anti*-CH_3_CHOO + *anti*-CH_3_CHOO = *anti*-CH_3_CHOO-*anti*-CH_3_CHOO (LM1), the variational TST was used to compute their association and dissociation rate constants. The RRKM theory was used to study the effect of pressure on the formation and quenching of excited intermediates. Kinetic calculations were carried out by using the Variflex code.^15^

## 3 Results and Discussions

### 3.1 Heats of Formation of CH_3_CHOO Conformers

The heats of formation of both *syn-* and *anti-*CH_3_CHOO conformers have been evaluated at the CCSDTQ/CBS­(D,T,Q,5,6)+Δ// CCSD­(T)/ANO2 level of theory by Begley et al.^16^ to be 12.2 and 15.6 kcal mol^–1^, respectively. The results agree closely with the recently reported values by Ruscic and Bross, 12.25 and 15.70 kcal mol^–1^ at 0 K, listed in the Active Thermochemical Tables (ATcT).^17^

We employed the isodesmic nature of the dative bond exchange in the N_2_ reaction,^18^ represented by CH_3_CHO →O + N_2_ **=** CH_3_CHO + N_2_ → O, for estimation of the heats of formation of CH_3_CHOO conformers, using the reliably predicted heats of the reaction (Δ_r_ *H*°) and the experimentally well-established heats of formation of CH_3_CHO and N_2_O.^17^ The energy balance of the reaction gives Δ_f_ *H*° (CH_3_CHOO) = Δ_r_ *H*° + Δ_f_ *H*° (CH_3_CHO) + Δ_f_ *H*° (N_2_O) at 0 K. Based on the values of Δ_r_ *H*° predicted at 2 different levels of theory, we obtained: I, at the CCSD­(T)/aug-cc-pVTZ//B3LYP/aug-cc-pVTZ level, 11.82 and 15.31 kcal mol^–1^ for *syn*-CH_3_CHOO and *anti*-CH_3_CHOO respectively; and II, at the CCSD­(T)/CBS­(T,Q,5)//B3LYP/aug-cc-pVTZ level, 12.71 and 16.18 kcal mol^–1^ for *syn*-CH_3_CHOO and *anti*-CH_3_CHOO, respectively, as listed in Table 1. The isodesmic characteristics of dative bond exchange in the N_2_ reaction provide reliable estimates of heats of formation by canceling out isodesmic errors, though they may not be applicable for estimating reaction barriers. Our results are consistent with the values of Begley et al.^16^ and of Ruscic and Bross^17^ within ±0.5 kcal mol^–1^ as summarized in Table 1.

**Table 1:** Comparison of the Heats of Formation of CH_3_CHOO Conformers Predicted by Different Authors (in kcal mol^–1^ at 0 K)

	this work^a^		literature
Δ_f_ *H*°_0_	I	II	
*syn*-CH_3_CHOO	11.82	12.71	12.2^b^; 12.25 ± 0.13^c^
*anti*-CH_3_CHOO	15.31	16.18	15.6^b^; 15.70 ± 0.15^c^

a I, CCSD­(T)/aug-cc-pVTZ//B3LYP/aug-cc-pVTZ; and II, CCSD­(T)/CBS­(T,Q,5)//B3LYP/aug-cc-pVTZ.

b CCSDTQ/CBS­(D,T,Q,5,6)+Δ//CCSD­(T)/ANO2 (ref 16).

c Ref 17.

### 3.2 Potential Energy Surfaces and the Mechanism of the CH_3_CHOO + CH_3_CHOO Reactions

#### 3.2.1 The *anti*-CH_3_CHOO + *anti*-CH_3_CHOO Reaction

Figure 1 presents the predicted PES of the *anti*-CH_3_CHOO + *anti*-CH_3_CHOO reaction in the gas phase computed at the CCSD­(T)/aug-cc-pVTZ//B3LYP/aug-cc-pVTZ level of theory. The geometries optimized at the B3LYP/aug-cc-pVTZ level for various species are presented in Figure S1, while the vibrational frequencies and moments of inertia (*I*_A_, *I*_B_, *I*_C_) for reactants, intermediates, transition states, and products are summarized in Table S1 in the Supporting Information section.

**Figure 1:**
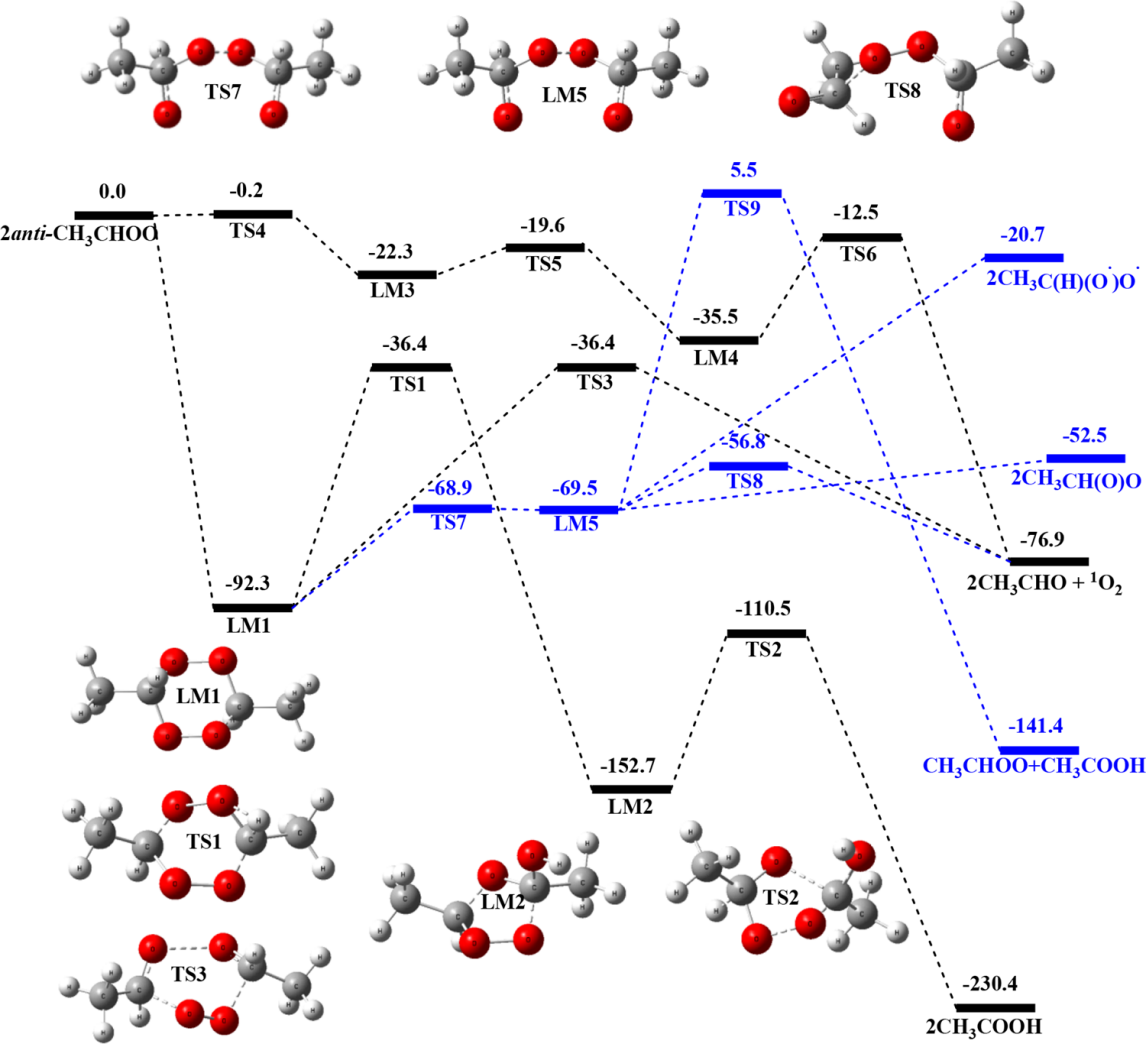
Potential energy profile of the *anti*-CH_3_CHOO + *anti*-CH_3_CHOO reaction computed at the CCSD­(T)/aug-cc-pVTZ//B3LYP/aug-cc-pVTZ level (energy in kcal mol^–1^). The blue reaction paths represent the steps deriving from the ring-opening channel.

The first lower energy product channel occurs by the initial formation of a 6-membered-ring complex of *anti*-CH_3_CHOO**···** *anti*-CH_3_CHOO intermediate LM1, in which the terminal O atom of one of the CH_3_CHOO molecules binds with the C atom of the CH group of the second CH_3_CHOO molecule, producing half of the ring. The second half of the ring is formed in a similar manner reversing the role of the CH_3_CHOO molecules (see Figure S1), with the binding energy of 92.3 kcal mol^–1^. LM1 can undergo intramolecular H transfer from one of the CH groups to its neighboring O atom via TS1, with the energy barrier of 55.9 kcal mol^–1^ above LM1 forming the 5-membered-ring intermediate LM2, lying 152.7 kcal mol^–1^ below the reactants (see Figure S1). A similar H transfer process involving the second half of the ring in LM2 via TS2 can produce 2 CH_3_COOH, releasing 230.4 kcal mol^–1^ of energy. The second lower energy pathway from LM1 occurs via a 6-membered-ring TS3 (see Figure S1) by breaking the C–O bonds to eliminate O_2_ with the energy barrier of 55.9 kcal mol^–1^ above LM1, giving 2 CH_3_CHO + O_2_(^1^Δ), denoted as ^1^O_2_ below, and releases 76.9 kcal mol^–1^ of energy. Another pathway can also form 2 CH_3_CHO + ^1^O_2_ without involving LM1, but with a higher energy path via TS4. In this process, the C atom of the CH group of one of the CH_3_CHOO molecules binds with the C atom of the CH group of another CH_3_CHOO molecule with a slightly negative energy of 0.2 kcal mol^–1^ above the reactants to form the complex LM3, with the binding energy of 22.3 kcal mol^–1^. LM3 can isomerize via a 6-membered-ring transition state, TS5 (See Figure S1), in which the C atom of the CH group of one of the CH_3_CHOO molecules binds with the C atom of the CH group of another CH_3_CHOO molecule and the terminal O atom of one of the CH_3_CHOO molecules binds with the terminal O atom of another CH_3_CHOO molecule with the energy barrier of 2.7 kcal mol^–1^ above LM3 to form of a 6-membered-ring complex LM4. LM4 can undergo the O_2_ elimination reaction via TS6 producing the final products, 2 CH_3_CHO + ^1^O_2_, as shown in Figure 1.

There is an additional pathway from LM1 where one of the O–O bonds breaks through the transition state TS7, which has an energy of −68.9 kcal/mol. This process results in the formation of the diradical LM5, CH_3_CH­(O^·^)­OOCH­(O^·^)­CH_3_, which has an energy of −69.5 kcal/mol, suggesting that the LM5 is unstable as has also been shown by Vereecken et al.^5^ for the CH_2_OO dimer case. From LM5, there is a transition state, TS8, with an energy of −56.8 kcal/mol, producing 2CH_3_CHO + ^1^O_2_. This mechanism is similar to the self-reaction of CH_2_OO reported by Vereecken et al.^5^ as mentioned above. Additionally, there are other products (CH_3_CHOO + CH_3_COOH) formed via transition state TS9, which has a much higher energy of 5.5 kcal/mol. This process involving H-transfer is, therefore, kinetically unimportant.

#### 3.2.2 The *anti*-CH_3_CHOO + *syn*-CH_3_CHOO Reaction

Figure 2 presents the predicted PES of the *anti*-CH_3_CHOO + *syn*-CH_3_CHOO reaction computed at the CCSD­(T)/aug-cc-pVTZ//B3LYP/aug-cc-pVTZ level of theory. The geometries optimized at the B3LYP/aug-cc-pVTZ level for various species are presented in Figure S2, while the vibrational frequencies and moments of inertia (*I*_A_, *I*_B_, *I*_C_) for reactants, intermediates, transition states, and products are summarized in Table S2 in the Supporting Information section. The lower energy product channel can occur by the initial formation of the 6-membered-ring *anti*-CH_3_CHOO**···** *syn*-CH_3_CHOO intermediate LM1’, similar to the aforementioned mechanism for the LM1 formation, with a binding energy of 82.7 kcal mol^–1^. LM1’ can fragment via the 6-membered-ring transition state, TS3′, similar to TS3 in the previous reaction, by breaking the C–O bonds and eliminating O_2_, with the activation energy of 51.7 kcal mol^–1^ above LM1’ yielding 2 CH_3_CHO + ^1^O_2_ and releasing 73.4 kcal mol^–1^ of energy. Another pathway that also produces 2 CH_3_CHO + ^1^O_2_ involves a higher energy 5-membered-ring intermediate LM2’, in which the C atom of the CH group of one of the CH_3_CHOO molecules binds with the central O atom of another CH_3_CHOO molecule with the binding energy of 37.5 kcal mol^–1^ above the reactants, similar to the formation of LM2 mentioned above. LM2’ then can decompose via 5-membered-ring transition state TS2’ (see Figure S2) with an energy barrier of 13.5 kcal mol^–1^ giving the cited products, as shown in Figure 2.

**Figure 2:**
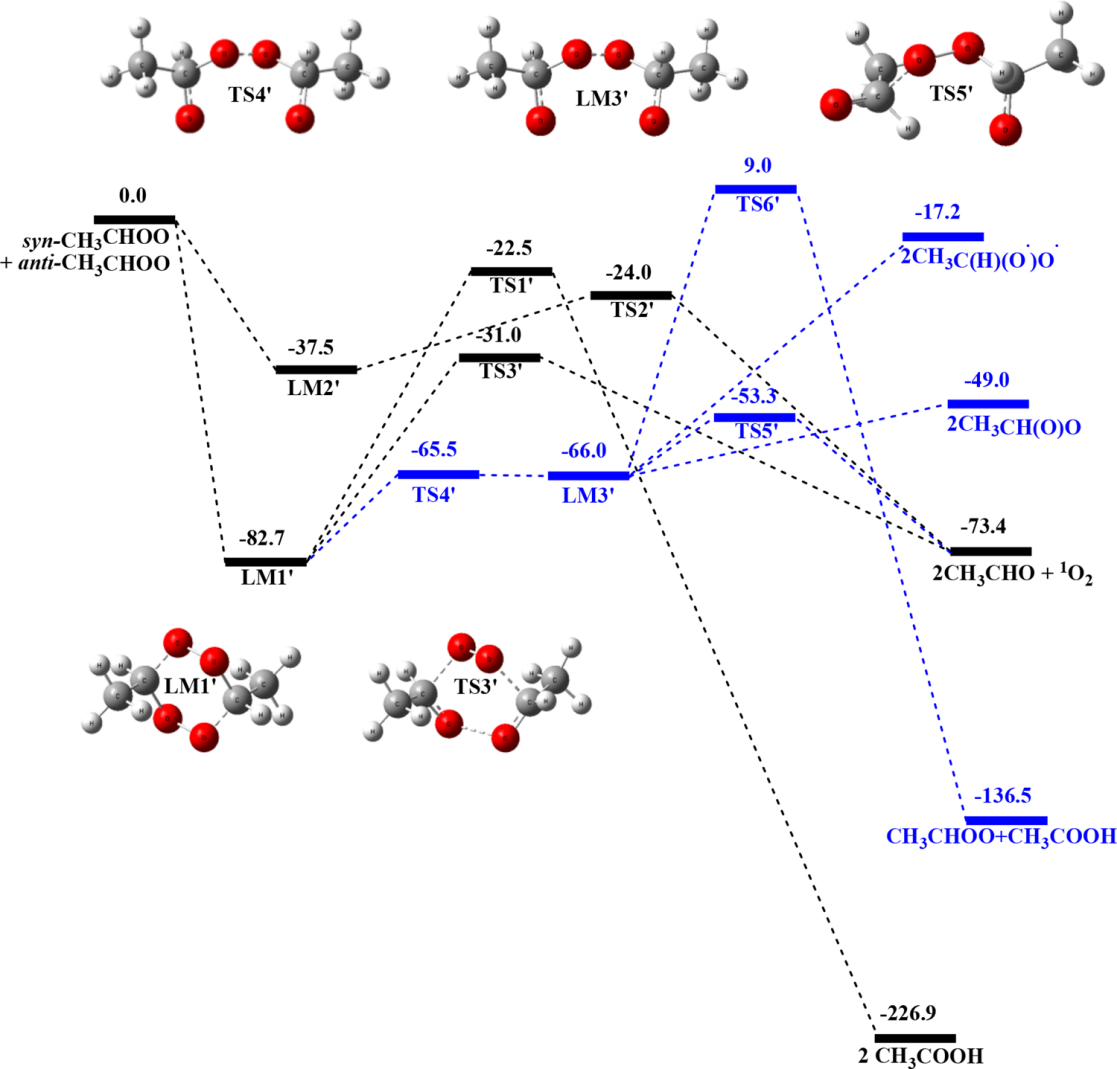
Potential energy profile of the *anti*-CH_3_CHOO + *syn*-CH_3_CHOO reaction computed at the CCSD­(T)/aug-cc-pVTZ//B3LYP/aug-cc-pVTZ level (energy in kcal mol^–1^). The blue reaction paths represent the steps deriving from the ring-opening channel.

Similar to the *anti*-CH_3_CHOO + *anti*-CH_3_CHOO reaction, there is an additional ring-opening pathway from LM1’ (−82.7 kcal/mol) through the transition state TS4’, which has an energy of −65.5 kcal/mol forming diradical LM3′ (−66.0 kcal/mol). The result again indicates that the diradical intermediate is unstable. From LM3′, there is another transition state, TS5′, with an energy of −53.3 kcal/mol, resulting in the formation of 2CH_3_CHO + ^1^O_2_. Additionally, there are other products (CH_3_CHOO + CH_3_COOH) formed via transition state TS6’ with a high energy of 9.0 kcal/mol, which is kinetically unimportant.

#### 3.2.3 The *syn*-CH_3_CHOO + *syn*-CH_3_CHOO Reaction

Figure 3 presents the predicted PES of the *syn*-CH_3_CHOO + *syn*-CH_3_CHOO reaction computed at the CCSD­(T)/aug-cc-pVTZ //B3LYP/aug-cc-pVTZ level of theory. The geometries optimized at the B3LYP/aug-cc-pVTZ level for various species are presented in Figure S3, while the vibrational frequencies and moments of inertia (*I*_A_, *I*_B_, I_C_) for reactants, intermediates, transition states, and products are summarized in Table S3 in the Supporting Information section. The reaction can occur by the 6-member-ring *syn*-CH_3_CHOO**···** *syn*-CH_3_CHOO intermediate LM1’’ with a binding energy of −9.8 kcal mol^–1^. LM1’’ can isomerize via the 6-membered-ring transition state, TS1’’, producing another 6-membered-ring intermediate LM2’’ (see Figure S3), in which the electronic structure is similar to the LM1 in the *anti-anti* reaction. LM2’’ can decompose via the 6-membered-ring transition state, TS2’’, whose electronic structure is similar to that of TS3 in the *anti-anti* reaction producing 2 CH_3_CHO + ^1^O_2_ with the release of 69.9 kcal mol^–1^ energy. Another lower energy path from LM2’’ can occur via the 7-membered-ring transition state TS3’’, in which the terminal O atom of one of the CH_3_CHOO molecules binds with the C atom of the CH group of the second CH_3_CHOO molecule, producing half of the ring. The second half of the ring involves the central O atom of one of the CH_3_CHOO molecules binding with the H atom of the CH_3_ group of the second CH_3_CHOO molecule (see Figure S3), with the binding energy of 70.0 kcal mol^–1^ above LM2’’, forming CH_3_CHO + CH_2_CHO + OOH and releasing 58.5 kcal mol^–1^ of energy. The third pathway from LM2’’ forms 2 CH_3_COOH, with the energy barrier of 60.3 kcal mol^–1^ at TS4’’ above the LM2’’, releasing a very large exothermicity of 223.4 kcal mol^–1^.

**Figure 3:**
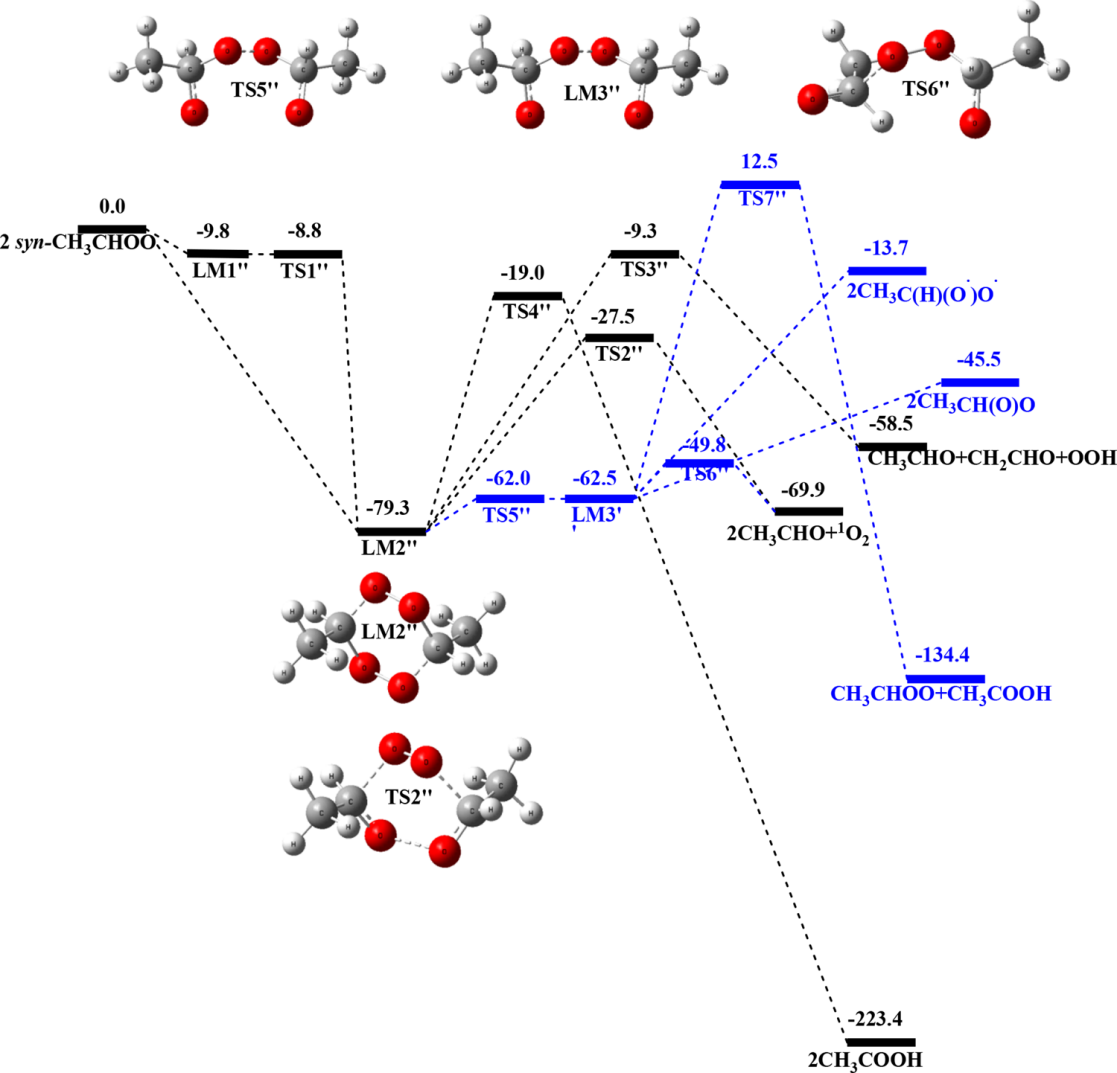
Potential energy profile of the *syn*-CH_3_CHOO + *syn*-CH_3_CHOO reaction computed at the CCSD­(T)/aug-cc-pVTZ//B3LYP/aug-cc-pVTZ level (energy in kcal mol^–1^). The blue reaction paths represent the steps deriving from the ring-opening channel.

Again, similar to the *anti*-CH_3_CHOO + *anti*-CH_3_CHOO reaction, there is an additional pathway from LM2’’ where one of the O–O bonds breaks through the transition state TS5’’, which has an energy of −62.0 kcal/mol. This process results in the formation of the diradical LM3″, which has an energy of −62.5 kcal/mol, suggesting again that LM3″ is unstable, similar to the previous two cases. From LM3’’, there is a transition state, TS6’’, with an energy of −49.8 kcal/mol, resulting in the formation of 2CH_3_CHO + ^1^O_2_. Additionally, there are other products (CH_3_CHOO + CH_3_COOH) formed via transition state TS7’’ with a high energy of 12.5 kcal/mol, which is again kinetically unimportant.

### 3.3 Rate Constant Predictions

The kinetics for each reaction can be reliably computed with the Variflex code^15^ written on the basis of statistical TST and RRKM theories as aforementioned. For the initial association producing *anti*-CH_3_CHOO**···** *anti*-CH_3_CHOO, *anti*-CH_3_CHOO**···** *syn*-CH_3_CHOO, and *syn*-CH_3_CHOO**···** *syn*-CH_3_CHOO intermediates, the variational TST (VTST) based on the predicted MEPs was employed for rate constant calculations. Take the *anti*-CH_3_CHOO + *anti*-CH_3_CHOO association reaction, for example, its MEP was established by varying the 2 *anti*-CH_3_CHOO separation from 1.4 to 4.3 Å with a step size of 0.1 Å at the B3LYP/aug-cc-pVTZ level. The Morse function, $V(r)=D_{e}[(1-e^{-\beta (r-r_{e})}{)}^{2}-1]$ was utilized to represent the MEP obtained by full optimization along the varying reaction coordinate. Here, *D*_e_, *R* and *R*_e_ have the usual meanings. The predicted Morse function for the *anti*-CH_3_CHOO**···** *anti*-CH_3_CHOO → *anti*-CH_3_CHOO + *anti*-CH_3_CHOO MEP can be represented by β = 4.7 Å^–1^ with the values of *D*_e_ presented in Figure 1 (with the zero-point energy included in the MEP). Similarly, for the redissociation of the *anti*-CH_3_CHOO**···** *syn*-CH_3_CHOO, and *syn*-CH_3_CHOO**···** *syn*-CH_3_CHOO intermediates, their variational MEPs could be represented by the Morse functions with the β values, 2.8 and 2.6 Å^–1^, with the corresponding *D*_e_ values, respectively.

The key bimolecular association-decomposition mechanism for CH_3_CHOO conformers, as shown in Figures 1–3, can be represented by the following general scheme:

$${\mathrm{CH}}_{3}\mathrm{CHOO}+{\mathrm{CH}}_{3}\mathrm{CHOO}\to ({\mathrm{CH}}_{3}\mathrm{CHOO}{{)}_{2}}^{*}$$ 1

$$({\mathrm{CH}}_{3}\mathrm{CHOO}{{)}_{2}}^{*}\to \mathrm{products}$$ 2

$$({\mathrm{CH}}_{3}\mathrm{CHOO}{{)}_{2}}^{*}+M\to ({\mathrm{CH}}_{3}\mathrm{CHOO}{)}_{2}+M$$ 3

In the above scheme, * represents the internal excitation of the nascent dimer formed by the self- or cross-association reaction; M represents the third-body quencher such as He. As the concentrations of the CH_3_CHOO conformers were monitored in the experimental kinetic study of Kao et al.,^3^ the reported rate constants should include all product formation and the quenching reaction giving stabilized dimers. The collisional quenching rate was estimated with the exponential down model assuming <Δ*E*_down_> = 70 cm^–1^,^19^ for the He-dimer collisional energy transfer with the Lennard-Jones collision frequency computed by the L-J potential predicted at the B3LYP/6–311+G­(3df,2p) level as shown in Figure S4, which gave rise to ε = 60.3 K and σ = 4.44 Å. Reaction step 2 in the general scheme includes 2–3 low-energy paths as shown in Figures 1–3.

The predicted temperature dependences of product formation rate constants, including the collisional quenching of internally excited association intermediates, are presented in Tables S3–S5 for the self- and cross-reaction of the two conformers. For the self-reaction of *anti*- CH_3_CHOO at 298 K under the 5–Torr He pressure, the collisional quenching of the excited dimer was found to be slightly faster than the formation of the major decomposition products, 2 CH_3_CHO + ^1^O_2_. The production of 2 CH_3_COOH was predicted to be <1% of CH_3_CHO (see Table S3). For the cross-reaction of the two conformers at 298 K under the 5–Torr He pressure, the quenching rate constant *k*_M_ was predicted to be about 4 times greater than that for the formation of the major products (2 CH_3_CHO + ^1^O_2_). The production of CH_3_COOH was again predicted to be of negligible importance (see Table S4). Finally, for the self-reaction of *syn*-CH_3_CHOO at 298 K under the 5–Torr He pressure, the quenching rate constant *k*_M_ was found to be more than 2.5 times faster than that for the formation of the major products (2 CH_3_CHO + ^1^O_2_). CH_3_COOH and the HO_2_ radical product formation channels were found to be negligibly competitive.

Figure 4 graphically presents the theoretically predicted temperature-dependence of the rate constants listed in Tables S3–S5 for the self- and cross-reactions of *anti*- and *syn*-CH_3_CHOO conformers at 5–Torr He. The channels producing 2 CH_3_CH­(O)O and 2CH_3_C­(H)­(O^·^)­(O^·^) are not included in the kinetic calculations on account of their high energies. The total reaction rate constants are noted to be steadily increasing with temperature. At 298 K under the 5–Torr He pressure, the predicted rate constants for these reactions can be given, respectively, by *k*_aa_ = 4.90 × 10^–10^ cm^3^ molecule^–1^ s^–1^, *k*_as_ = 1.82 × 10^–10^ cm^3^ molecule^–1^ s^–1^, and *k*_ss_ = 1.28 × 10^–10^ cm^3^ molecule^–1^ s^–1^. The theoretical kinetic results agree with the experimental data provided by Kao et al.,^3^ within their experimental errors as shown in the figure and in Table 2. In addition, our study indicates that *k*_aa_ > *k*_ss_, is also consistent with the finding of Sheps et al.^7^ The predicted rate constants for key product formation are presented in the SI section (Tables S3–S5), showing the key product ratios, acetaldehyde vs acetic acid. The results indicate that acetaldehyde is dominant over acetic acid by as much as 4:1 under the condition studied by Kao et al.^3^

**Figure 4:**
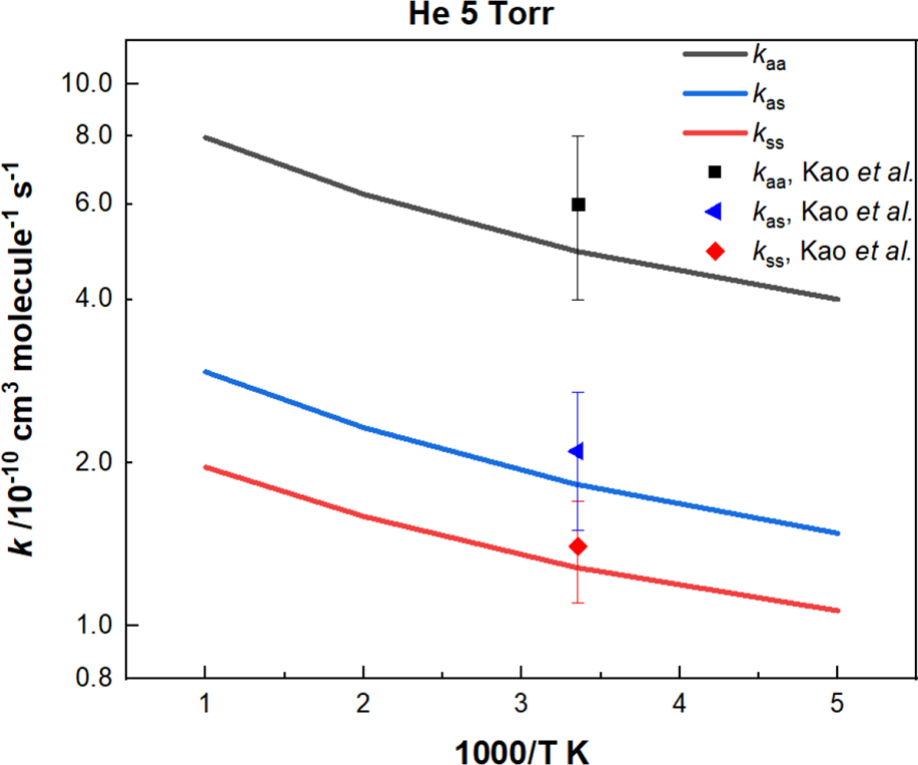
Predicted rate constants for the self- and cross-reactions of CH_3_CHOO conformers comparing with the experimental results of Kao et al. (ref 3).

**Table 2:** Predicted Bimolecular Decay Rate Constants (in Units of 10^–10^ cm^3^ molecule^–1^ s^–1^) for *anti*-CH_3_CHOO, *syn*-CH_3_CHOO and CH_2_OO Comparing with Available Experimental Results

	rate constant		
reactions	theory	experiment	products
*anti*-CH_3_CHOO + *anti*-CH_3_CHOO	4.90	6.00 ± 2^b^	2 CH_3_CHO + ^1^O_2_, 2 CH_3_COOH, (CH_3_CHOO)_2_
*anti*-CH_3_CHOO + *syn*-CH_3_CHOO	1.82	2.10 ± 0.6^b^	2 CH_3_CHO + ^1^O_2_, (CH_3_CHOO)_2_
*syn*-CH_3_CHOO + *syn*-CH_3_CHOO	1.28	1.40 ± 0.3^b^	2 CH_3_CHO + ^1^O_2_, 2 CH_3_COOH, CH_3_CHO + CH_2_CHO + OOH, (CH_3_CHOO)_2_
CH_2_OO + CH_2_OO	4.00 ± 2.0^a^	4.10 ± 0.8^a^	2 CH_2_O + ^1^O_2_

a Su et al.^4^

b Kao et al.^3^

In Table 2, we also list the result for the bimolecular self-reaction of CH_2_OO previously reported by Su et al.,^4^ revealing the zwitter-ionic effect on the bimolecular CI reactions.

## 4 Conclusions

The self- and cross-reaction mechanisms of *anti*-CH_3_CHOO and *syn*-CH_3_CHOO conformers were studied at the CCSD­(T)/aug-cc-pVTZ level of theory based on the geometries of all species involved, computed with the B3LYP/aug-cc-pVTZ method. The results of our study carried out at 298 K under 5–Torr He pressure give *k*_aa_ = 4.90 × 10^–10^ cm^3^ molecule^–1^ s^–1^ for the self-reaction of *anti*-CH_3_CHOO, *k*_as_ = 1.82 × 10^–10^ cm^3^ molecule^–1^ s^–1^ for the *anti-syn* cross-reaction, and *k*_ss_ = 1.28 × 10^–10^ cm^3^ molecule^–1^ s^–1^ for the self-reaction of *syn*-CH_3_CHOO, fully consistent with the experimentally observed trend: *k*_aa_ > *k*_as_ > *k*_ss_.^3,7^ The predicted absolute rate constants at 298 K under 5–Torr He pressure agree closely with the experimental values within reported errors measured at 298 K under 2–10 Torr He pressure by Lee and co-workers.^3^ The predicted values include the collisional quenching and fragmentation of internally excited dimers formed by the initial association reaction; the collisional quenching process accounts for more than 50% of the measured CH_3_CHOO conformer decay rates.

## Supplementary Material



## Notes

Details of electronic structures, vibrational frequencies, moments of inertia of all the species involved in the *anti*-CH_3_CHOO + *anti*-CH_3_CHOO, *anti*-CH_3_CHOO + *syn*-CH_3_CHOO, and *syn*-CH_3_CHOO + *syn*-CH_3_CHOO reactions, as well as the Lennard-Jones potential, and predicted reaction rates (PDF)
