# Supplementary material for: Ab Initio Chemical Kinetics for Self- and Cross-Reactions of anti- and syn-CH3CHOO Conformers
Source: J Phys Chem A. 2026 Mar 17;130(12):2595–601. doi: 10.1021/acs.jpca.6c00127 (PMC13034461; doi:10.1021/acs.jpca.6c00127)
Supplement: Supplementary file 1 [file jp6c00127_si_001.pdf]

Supporting Information

Ab initio Chemical Kinetics for Self- and Cross-reactions of *anti*- and *syn*-CH_3CHOO Conformers

Hue-Phuong Trac, Putikam Raghunath and Ming-Chang Lin*

Department of Applied Chemistry and Center for Emergent Functional Matter Science, National Yang Ming Chiao Tung University, Hsinchu 300093, Taiwan.

***Corresponding authors:**

M. C. Lin, email address: chemmcl@emory.edu

ORCID: 0000-0003-3963-6017

Potential energy surfaces and the mechanism of the $CH_3CHOO + CH_3CHOO$ reaction

1. The *anti*-CH_3CHOO + *anti*-CH_3CHOO reaction

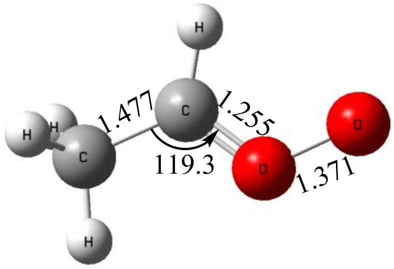

anti-CH_3CHOO

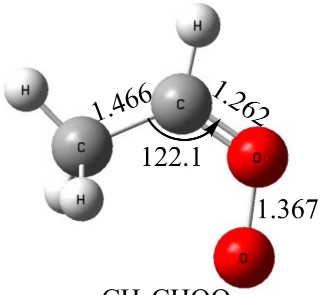

syn-CH_3CHOO

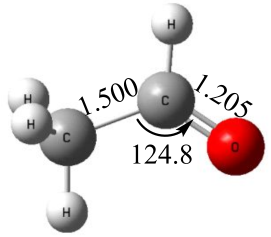

CH_3CHO

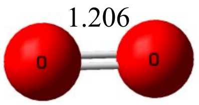

O_2

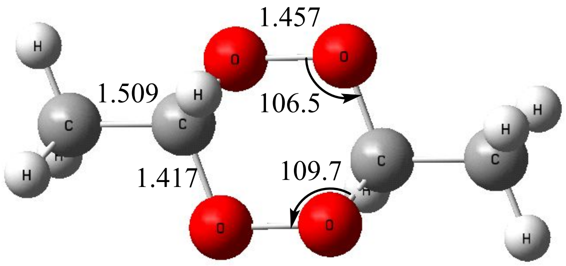

LM1

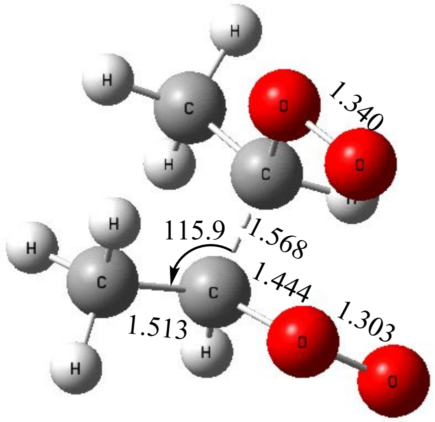

LM3

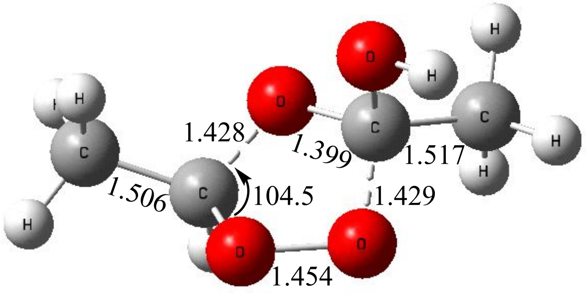

LM2

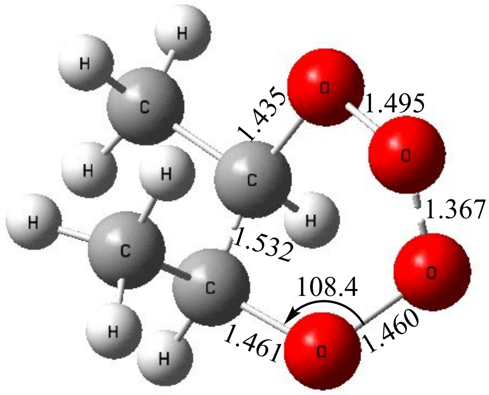

LM4

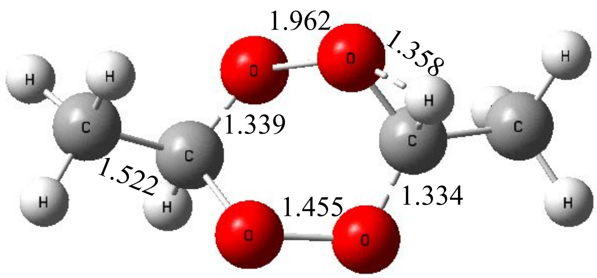

TS1

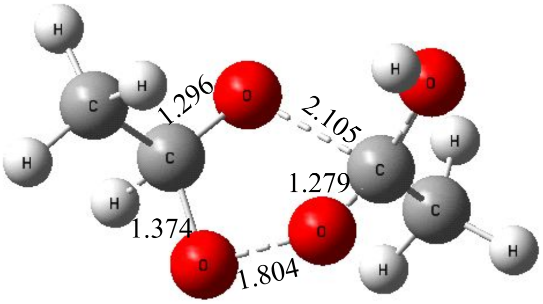

TS2

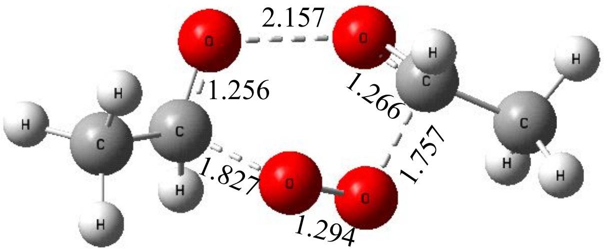

TS3

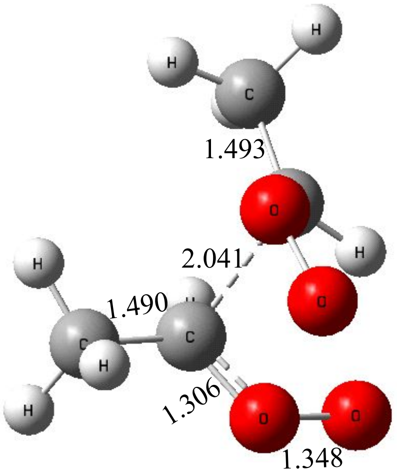

TS4

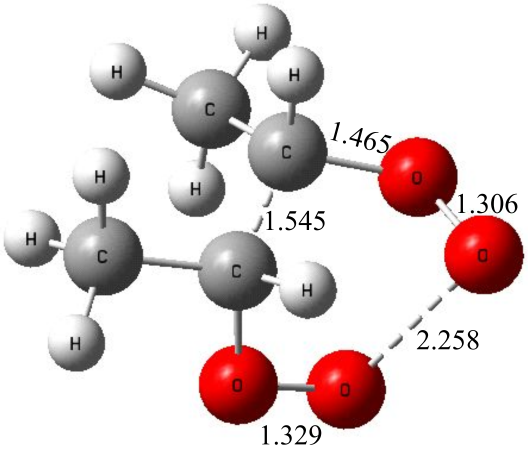

TS5

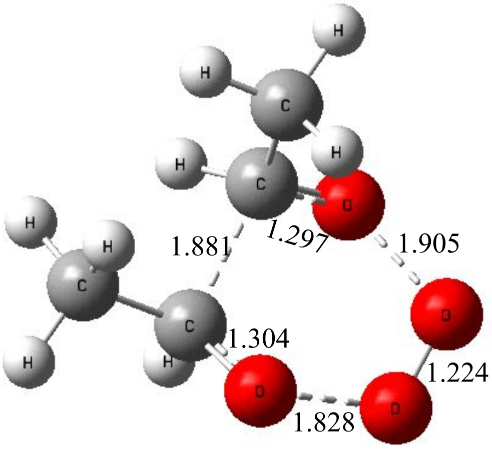

TS6

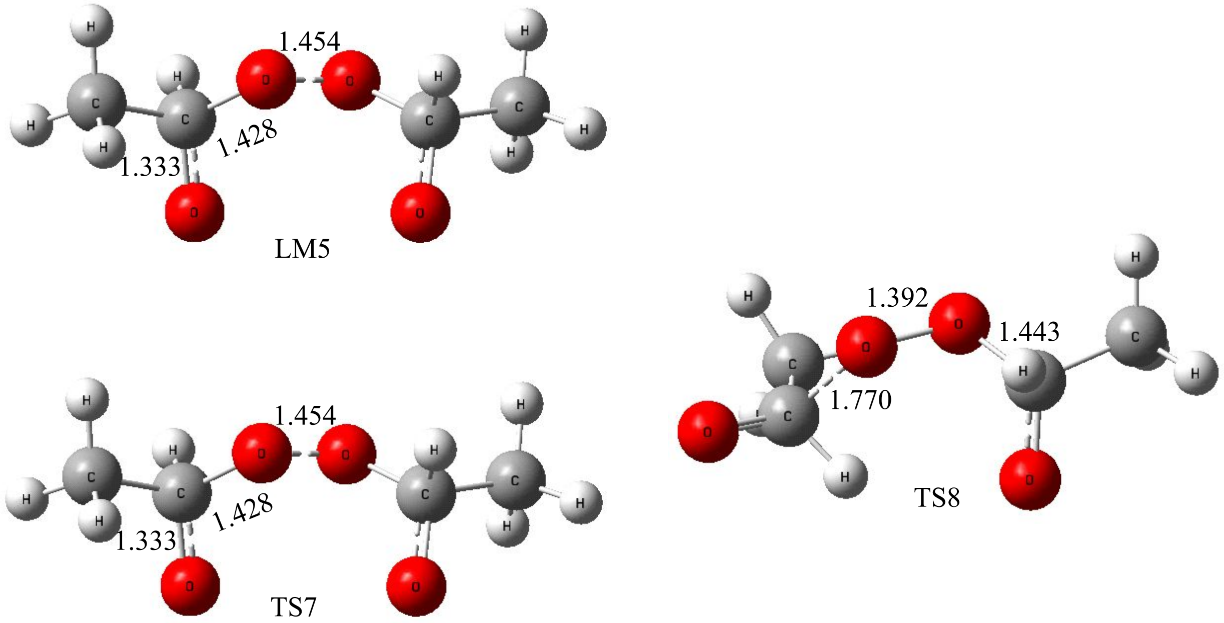

Figure S1. Geometries of reactants, intermediates, transition states and products of the reaction *anti*-CH_3CHOO + *anti*-CH_3CHOO computed at the B3LYP/aug-cc-pVTZ level. Bond lengths and bond angles are in angstroms (Å) and degrees (°), respectively.

Table S1. Vibrational frequencies and moments of inertia (I_A, I_B, I_C) for reactants, intermediates, transition states, and products of the *anti*-CH_3CHOO + *anti*-CH_3CHOO computed at the B3LYP/Aug-cc-PVTZ level.

Species or Transition states	Moments of inertia I_A, I_B, I_C (a.u)	Vibrational Frequencies (cm^{-1})
anti-CH_3CHOO	36.2, 408.1, 433.2	160, 258, 324, 554, 868, 893, 968, 1067, 1157, 1344, 1415, 1459, 1463, 1577, 3020, 3062, 3132, 3146
LM1	364.5, 1164.7, 1436.2	135, 180, 183, 264, 299, 300, 344, 457, 499, 560, 596, 721, 851, 855, 867, 886, 917, 949, 1073, 1091, 1110, 1165, 1170, 1177, 1361, 1364, 1372, 1377, 1411, 1416, 1483, 1483, 1490, 1492, 3048, 3050, 3052, 3052, 3126, 3127, 3128, 3128
LM2	458.7, 1096.6, 1151.3	73, 199, 205, 219, 246, 271, 367, 378, 499, 511, 572, 656, 696, 827, 863, 889, 902, 930, 968, 1086, 1125, 1132, 1156, 1181, 1216, 1324, 1375, 1400, 1414, 1427, 1483, 1486, 1487, 1493, 2997, 3051, 3053, 3119, 3123, 3134, 3143, 3803

LM3	687.1, 982.7, 1146.3	91, 165, 182, 196, 226, 250, 281, 335, 378, 464, 548, 588, 652, 747, 810, 898, 989, 1017, 1021, 1089, 1129, 1144, 1175, 1194, 1274, 1300, 1359, 1375, 1420, 1422, 1487, 1488, 1500, 1506, 3023, 3048, 3055, 3096, 3114, 3116, 3120, 3136
LM4	630.7, 854.2, 1142.9	76, 154, 207, 227, 271, 321, 380, 427, 471, 533, 602, 669, 739, 770, 806, 890, 899, 954, 988, 1018, 1086, 1123, 1158, 1196, 1321, 1353, 1378, 1396, 1413, 1423, 1485, 1490, 1497, 1508, 3022, 3033, 3043, 3046, 3105, 3109, 3126, 3128
LM5	374.8, 1670.8, 1752.6	45, 55, 98, 181, 182, 203, 287, 309, 352, 410, 504, 579, 580, 829, 847, 908, 909, 945, 970, 985, 1048, 1049, 1130, 1179, 1183, 1187, 1277, 1286, 1388, 1388, 1479, 1479, 1490, 1491, 2866, 2866, 3060, 3060, 3139, 3139, 3147, 3147
TS1	411.1, 1167.8, 1303.8	80,137,163,214,274, 350, 363, 399, 456, 507, 549, 581, 670, 726, 812, 863, 891, 920, 1021, 1040, 1054, 1122, 1172, 1251, 1282, 1376, 1394, 1397, 1433, 1473, 1479, 1483, 1490, 1742, 2902, 3041, 3053, 3108, 3124, 3126, 3154, 1350*i*
TS2	523.1, 1134.6, 1204.8	96, 138, 168, 187, 213, 255, 317, 422, 437, 498, 540, 569, 619, 671, 724, 860, 882, 983, 1018, 1030, 1052, 1108, 1157, 1195, 1347, 1357, 1387, 1405, 1465, 1479, 1482, 1482, 1524, 2422, 3038, 3046, 3118, 3129, 3134, 3183, 3742, 723*i*
TS3	390.7, 1419.7, 1704.8	26, 112, 139, 166, 170,175, 232, 288, 308, 430, 458, 486, 523, 533, 875, 897, 980, 983, 1074, 1089, 1100, 1162, 1210, 1338, 1351, 1370, 1375, 1426, 1443,1468, 1472, 1489, 1501, 2976, 2981, 3040, 3041, 3112, 3113, 3139, 3141, 524*i*
TS4	798.0, 1127.1, 1167.0	67, 130, 151, 169, 175, 226, 304, 307, 347, 399, 481, 567, 581, 858, 916, 933, 967, 998, 1048, 1100, 1146, 1151, 1171, 1275, 1305, 1394, 1410, 1426, 1441, 1476, 1479, 1492, 1498, 2962, 3032, 3035, 3093, 3099, 3125, 3127, 3139, 371*i*
TS5	691.0, 908.4, 1160.3	123, 187, 197, 233, 279, 311, 353, 379, 431, 513, 559, 685, 750, 832, 867, 977, 999, 1006, 1102, 1117, 1153, 1161, 1204, 1293, 1318, 1341, 1397, 1419, 1423, 1487, 1488, 1498, 1506, 3035, 3047, 3052, 3067, 3113, 3114, 3120, 3129, 266*i*
TS6	704.2, 926.2, 1347.1	100, 116, 117, 199, 237, 273, 281, 314, 405, 484, 507, 536, 575, 607, 808, 913, 952, 1012, 1037, 1064, 1112, 1193, 1263, 1292, 1327, 1377, 1394, 1404, 1405, 1473, 1484, 1493, 1499, 2977, 2989, 3040, 3044, 3102, 3107, 3121, 3137,733*i*

TS7	385.9, 1578.5, 1834.2	55, 74, 179, 203, 224, 276, 334, 371, 441, 478, 536, 606, 817, 830, 908, 912, 931, 955, 972, 1044, 1075, 1098, 1147, 1182, 1186, 1281, 1302, 1383, 1387, 1475, 1478, 1487, 1490, 2893, 2955, 3057, 3060, 3139, 3140, 3146, 3147, 56*i*
TS8	417.8, 1743.0, 1848.0	55, 59, 69, 165, 183, 226, 273, 314, 382, 419, 476, 509, 580, 805, 830, 898, 909, 939, 973, 1039, 1048, 1093, 1142, 1181, 1203, 1273, 1381, 1390, 1459, 1478, 1481, 1489, 1490, 2746, 2892, 3051, 3061, 3119, 3140, 3149, 3152, 784 *i*
$^1O_2$	0.0, 41.5, 41.5	1614.8
CH_3CHO	31.4, 177.5, 197.8	158, 510, 775, 886, 1129, 1136, 1379, 1422, 1460, 1469, 1805, 2870, 3022, 3073, 3135
$CH_3C(O)OH$	158.9, 190.5, 338.4	56, 424, 546, 584, 663, 858, 999, 1070, 1201, 1335, 1409, 1472, 1478, 1812, 3053, 3109, 3160, 3738

2. The *anti*-CH_3CHOO + *syn*-CH_3CHOO reaction

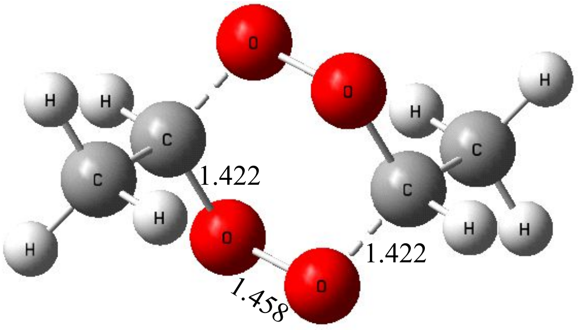

LM1'

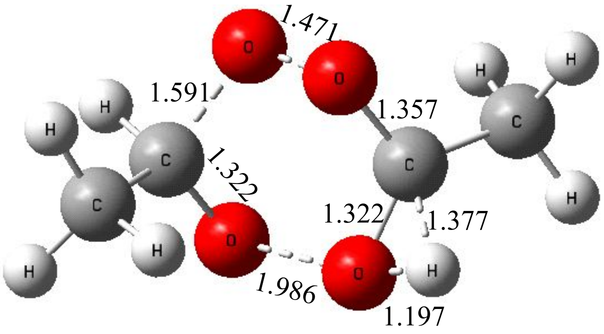

TS1'

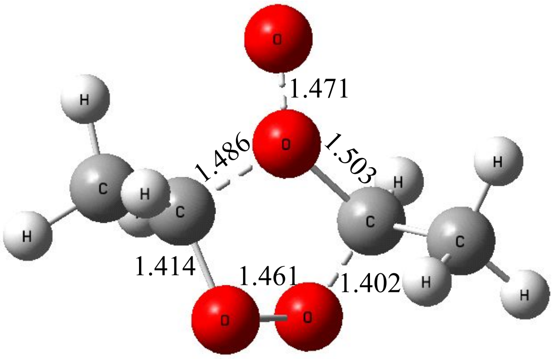

LM2'

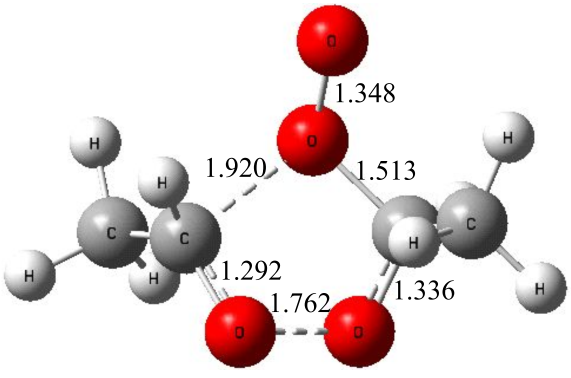

TS2'

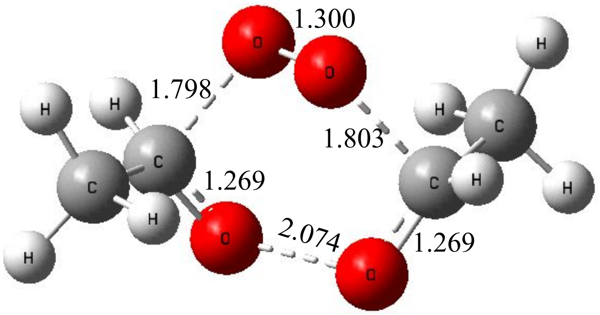

TS3'

Figure S2. Geometries of reactants, intermediates, transition states and products of the reaction *anti*-CH_3CHOO + *syn*-CH_3CHOO computed at the B3LYP/aug-cc-pVTZ level. Bond lengths and bond angles are in angstroms (Å) and degrees (°), respectively.

Table S2. Vibrational frequencies and moments of inertia (I_A, I_B, I_C) for reactants, intermediates, transition states, and products of the *anti*-CH_3CHOO + *syn*-CH_3CHOO computed at the B3LYP/Aug-cc-PVTZ level.

Species or Transition states	Moments of inertia I_A, I_B, I_C (a.u)	Vibrational Frequencies (cm^{-1})
syn-CH_3CHOO	101.0, 256.2, 346.5	188, 296, 462, 675, 758, 903, 983, 1047, 1111, 1349, 1400, 1438, 1461, 1559, 3015, 3049, 3140, 3179
LM1'	493.9, 932.0, 1073.6	160, 166, 173, 199, 263, 365, 463, 480, 507, 540, 668, 795, 818, 827, 837, 881, 900, 920, 1073, 1081, 1092, 1093, 1155, 1176, 1312, 1348, 1356, 1362, 1409, 1409, 1482, 1483, 1493, 1493, 3055, 3055, 3061, 3062, 3117, 3118, 3155, 3155
LM2'	596.3, 844.3, 1199.4	71, 167, 208, 231, 259, 303, 333, 413, 485, 526, 634, 653, 731, 812, 856, 863, 873, 902, 1047, 1064, 1102, 1110, 1152, 1161, 1265, 1286, 1377, 1390, 1409, 1420, 1472, 1485, 1486, 1487, 3051, 3057, 3108, 3120, 3127, 3135, 3144, 3160
TS1'	535.5, 1132.4, 1280.2	83, 102, 106, 153, 161, 189, 251, 311, 412, 437, 459, 475, 553, 583, 884, 900, 925, 958, 1023, 1081, 1116, 1124, 1229, 1270, 1283, 1392, 1395, 1427, 1447, 1478, 1481, 1482, 1484, 2969, 2971, 3043, 3043, 3109, 3109, 3139, 3140, 574*i*
TS2'	620.6, 933.5, 1263.9	83, 144, 178, 183, 232, 254, 294, 395, 441, 468, 496, 584, 620, 686, 872, 888, 942, 991, 1030, 1092, 1101, 1138, 1150, 1187, 1276, 1317, 1394, 1401, 1433, 1471, 1475, 1483, 1486, 2970, 3030, 3062, 3101, 3104, 3134, 3144, 3155, 467*i*
TS3'	505.6, 1058.8, 1191.2	82, 122,177, 182, 195, 277, 369, 407, 417, 456, 509, 534, 591, 608, 697, 831, 859, 890, 1008, 1024, 1039, 1127, 1209, 1225, 1271, 1343, 1372, 1389, 1397, 1461, 1471, 1484, 1487, 2091, 2932, 3025, 3047, 3100, 3108, 3135, 3154, 1393*i*

3. The *syn*-CH_3CHOO + *syn*-CH_3CHOO reaction

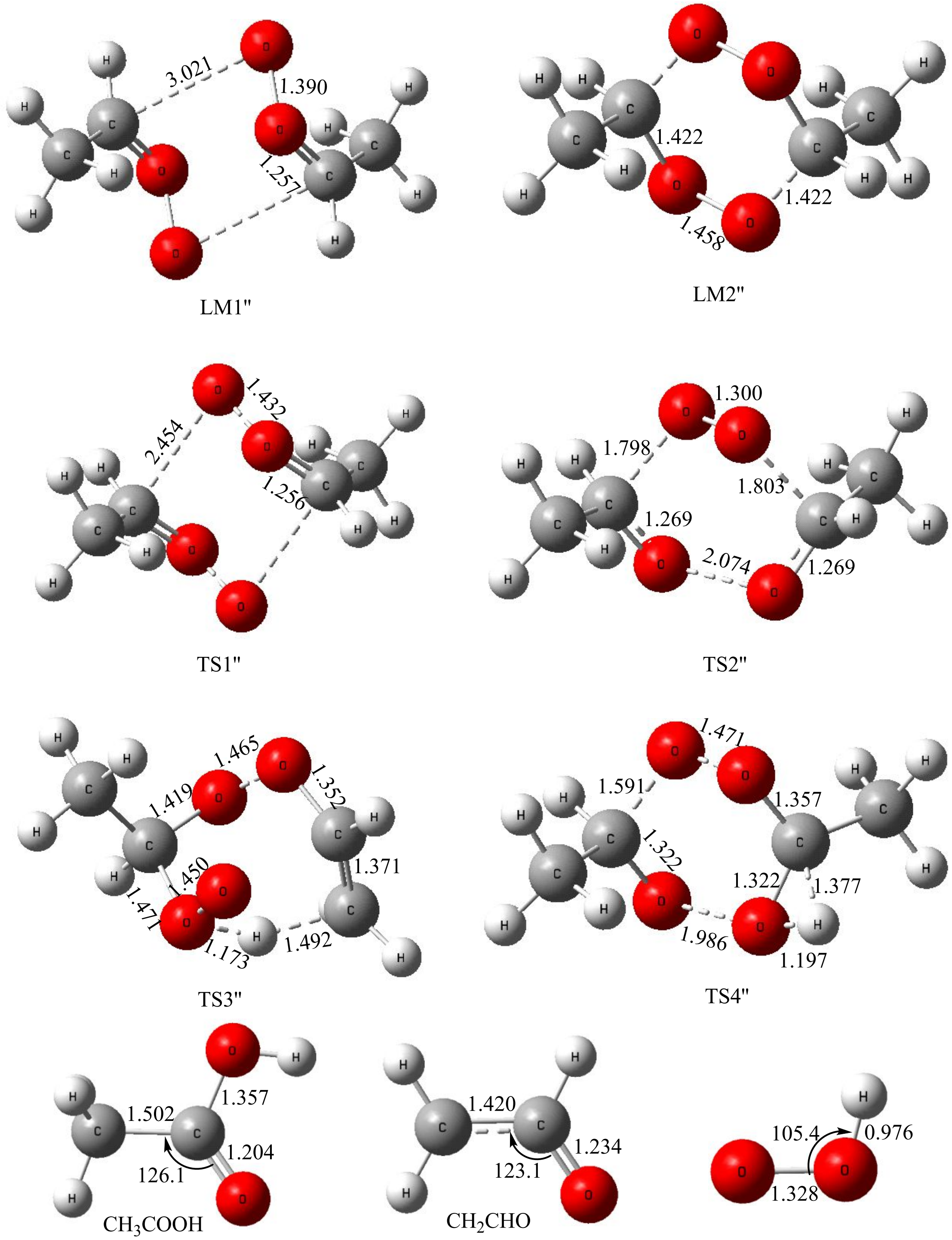

Figure S3. Geometries of reactants, intermediates, transition states and products of the reaction *syn*-CH_3CHOO + *syn*-CH_3CHOO computed at the B3LYP/aug-cc-pVTZ level. Bond lengths and bond angles are in angstroms (Å) and degrees (°), respectively.

Table S3. Vibrational frequencies and moments of inertia (I_A, I_B, I_C) for reactants, intermediates, transition states, and products of the *syn*-CH_3CHOO + *syn*-CH_3CHOO computed at the B3LYP/Aug-cc-PVTZ level.

Species or Transition states	Moments of inertia I_A, I_B, I_C (a.u)	Vibrational Frequencies (cm^{-1})
LM1''	643.8, 1305.7, 1576.1	28, 57, 58, 74, 85, 89, 149, 153, 294, 296, 452, 457, 665, 666, 762, 762, 852, 865, 987, 987, 1062, 1062, 1111, 1112, 1357, 1357, 1400, 1400, 1441, 1441, 1462, 1462, 1579, 1579, 3029, 3029, 3072, 3072, 3147, 3147, 3187, 3187
LM2''	493.9, 932.0, 1073.6	160, 166, 173, 199, 263, 365, 463, 480, 507, 540, 668, 795, 818, 827, 837, 881, 900, 920, 1073, 1081, 1092, 1093, 1155, 1176, 1312, 1348, 1356, 1362, 1409, 1409, 1482, 1483, 1493, 1493, 3055, 3055, 3061, 3062, 3117, 3118, 3155, 3155
TS1''	645.7, 1022.5, 1287.9	69, 95, 108, 147, 161, 177, 178, 311, 321, 383, 453, 627, 632, 789, 797, 822, 824, 949, 954, 1061, 1065, 1144, 1147, 1360, 1367, 1401, 1403, 1437, 1439, 1467, 1469, 1585,1590, 3011, 3011, 3121, 3121, 3168, 3169, 3186, 3187, 189*i*
TS2''	535.5, 1132.4, 1280.2	83, 102, 106, 153, 161, 189, 251, 311, 412, 437, 459, 475, 553, 583, 884, 900, 925, 958, 1023, 1081, 1116, 1124, 1229, 1270, 1283, 1392, 1395, 1427, 1447, 1478, 1481, 1482, 1484, 2969, 2971, 3043, 3043, 3109, 3109, 3139, 3140, 574*i*
TS3''	567.2, 899.0, 1079.8	149, 194, 246, 262, 344, 382, 416, 454, 480, 628, 631, 683, 756, 785, 819, 832, 871, 923, 942, 971, 1059, 1096, 1150, 1164, 1271, 1321, 1359, 1408, 1415, 1463, 1470, 1484, 1491, 1600, 3057, 3064, 3124, 3146, 3173, 3216, 3241, 1151*i*
TS4''	505.6, 1058.8, 1191.2	82, 122,177, 182, 195, 277, 369, 407, 417, 456, 509, 534, 591, 608, 697, 831, 859, 890, 1008, 1024, 1039, 1127, 1209, 1225, 1271, 1343, 1372, 1389, 1397, 1461, 1471, 1484, 1487, 2091, 2932, 3025, 3047, 3100, 3108, 3135, 3154, 1393*i*
CH_2CHO	26.7, 157.1, 183.8	446, 507, 768, 980, 982, 1161, 1398, 1475, 1545, 2944, 3142, 3253
OOH	2.9, 53.5, 56.4	1159, 1432, 3588

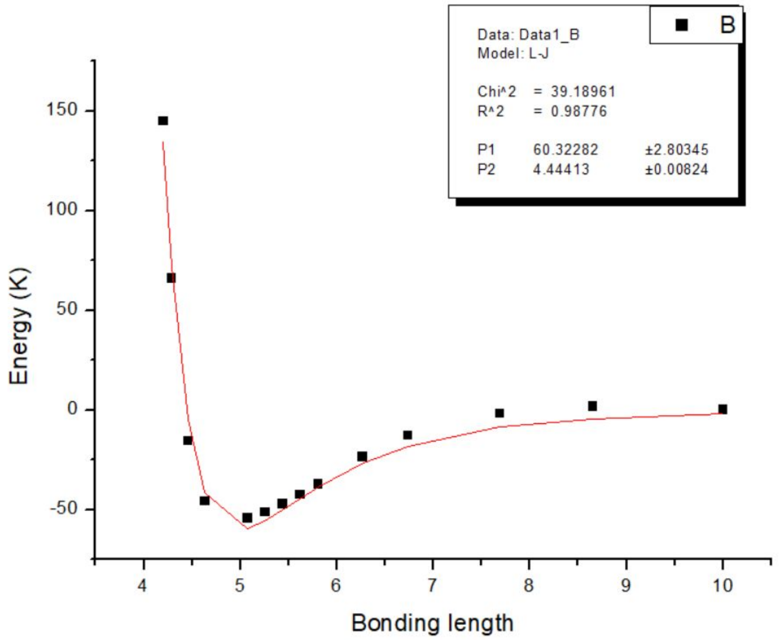

Fig. S4. The Lennard-Jones potential for the $(CH_3CHOO)_2$-He interaction computed at the B3LYP/6-311+G(3df,2p) level. Fitting of the cpomputed points to the L-J potential, $V(r) = 4\varepsilon [(\sigma/r)^{12} - (\sigma/r)^6]$, gave $\varepsilon = 60.3$ K and $\sigma = 4.44$ Å.

Table S4. Temperature dependence of predicted rate constants (in units of cm^3 $molecule^{-1}$ s^{-1}) in 5 Torr He bath gas for the self-reaction of *anti*-CH_3CHOO.

	Rate Constants				
T (K)	Dimer (k_M)	2 CH_3CHO + $^1O_2$	2 CH_3COOH	k_{total} (k_{aa})	CH_3CHO : CH_3COOH
200	2.00E-10	2.00E-10	1.16E-13	4.00E-10	99.9 : 0.1
298	2.45E-10	2.45E-10	1.90E-13	4.90E-10	99.9 : 0.1
300	2.46E-10	2.46E-10	1.92E-13	4.92E-10	99.9 : 0.1
500	3.13E-10	3.12E-10	5.05E-13	6.26E-10	99.8 : 0.2
1000	3.98E-10	3.94E-10	3.79E-12	7.96E-10	99.0 : 1.0

Table S5. Temperature dependence of predicted rate constants (in units of cm^3 $molecule^{-1}$ s^{-1}) in 5 Torr He bath gas for the cross-reaction of *anti*- and *syn*-CH_3CHOO conformers.

	Rate Constants				
T (K)	Dimer (k_M)	2 CH_3CHO + $^1O_2$	2 CH_3COOH	k_{total} (k_{as})	CH_3CHO : CH_3COOH
200	7.41E-11	7.41E-11	3.19E-15	1.48E-10	100.0 : 0.0
298	9.11E-11	9.11E-11	5.67E-15	1.82E-10	100.0 : 0.0
300	9.14E-11	9.14E-11	5.74E-15	1.83E-10	100.0 : 0.0
500	1.16E-10	1.16E-10	1.85E-14	2.32E-10	100.0 : 0.0
1000	1.47E-10	1.47E-10	2.13E-13	2.94E-10	99.9 : 0.1

Table S6. Temperature dependence of predicted rate constants (in units of cm^3 $molecule^{-1}$ s^{-1}) in 5 Torr He bath gas for the self-reaction of *syn*-CH_3CHOO.

	Rate Constants				
T (K)	Dimer (k_M)	2 CH_3CHO + $^1O_2$	2 CH_3COOH	k_{total} (k_{ss})	CH_3CHO : CH_3COOH
200	5.33E-11	5.33E-11	1.26E-15	1.07E-10	100.0 : 0.0
298	6.40E-11	6.40E-11	2.29E-15	1.28E-10	100.0 : 0.0
300	6.42E-11	6.42E-11	2.32E-15	1.28E-10	100.0 : 0.0
500	7.96E-11	7.96E-11	8.25E-15	1.59E-10	100.0 : 0.0
1000	9.81E-11	9.80E-11	1.13E-13	1.96E-10	99.9 : 0.1
